# Novel therapeutic ultrathin endoscope facilitates endoscopic intermuscular dissection for gastrointestinal stromal tumor adjacent to the anus

**DOI:** 10.1055/a-2779-7589

**Published:** 2026-02-09

**Authors:** Ting Li, Silin Huang, Suhuan Liao, Qiang Guo, Erzhen Zhong, Yudan Zhang, Haixia Wang

**Affiliations:** 1 Department of Gastroenterology, The First Peopleʼs Hospital of Yunnan Province, Affiliated Hospital of Kunming University of Science and Technology, Kunming, China; 2 Department of Gastroenterology, South China Hospital, Medical School, Shenzhen University, Shenzhen, China

A therapeutic ultrathin endoscope features a smaller outer diameter, greater bending capability, and a standard working channel, significantly improving the feasibility and efficiency of endoscopic submucosal dissection in challenging areas 1 2 . However, its application in the lower gastrointestinal tract remains unreported. Herein, we report a case in which a rectal subepithelial lesion (SEL) originating from the muscularis propria was successfully resected using endoscopic intermuscular dissection (EID) assisted using a therapeutic ultrathin endoscope (EG-840TP; Fujifilm, Tokyo, Japan).

**Fig. 1:**
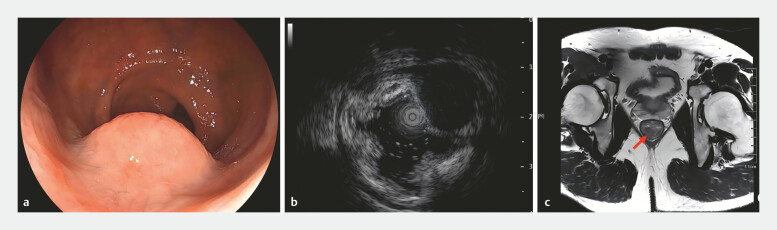
**a** A subepithelial lesion, measuring approximately 15 mm in diameter, was located in the distal rectum, adjacent to the anal verge. **b** EUS confirmed that the lesion originated from the muscularis propria. **c** MRI ruled out distant and lymph node metastasis. EUS, endoscopic ultrasound; MRI, magnetic resonance imaging.

**Fig. 2:**
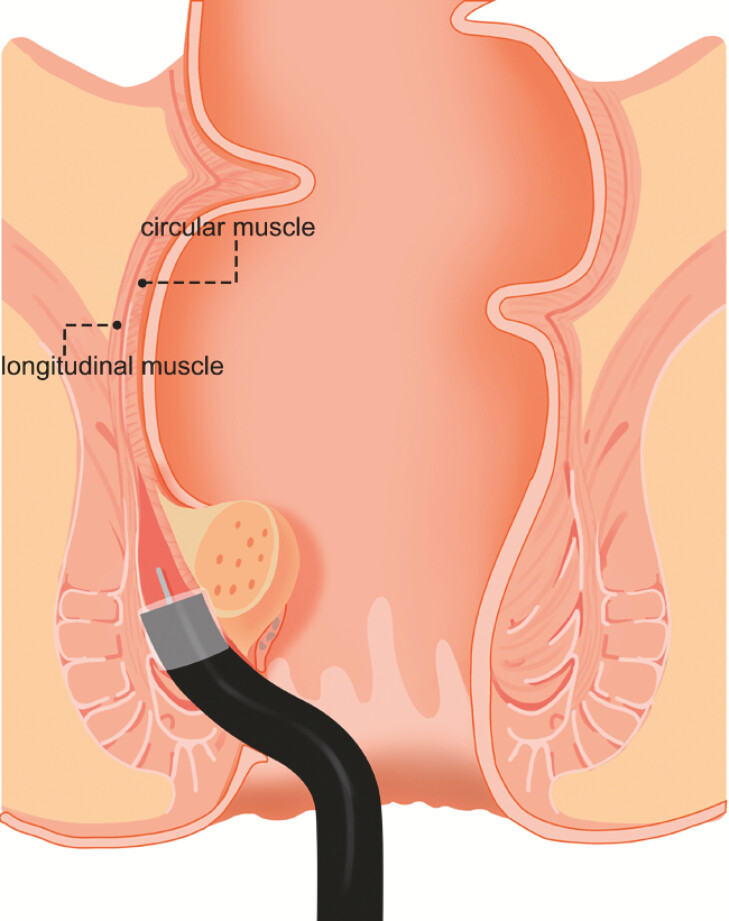
Schematic of the endoscopic intermuscular dissection strategy for the rectal subepithelial lesion utilizing a novel thin therapeutic endoscope.

**Fig. 3:**
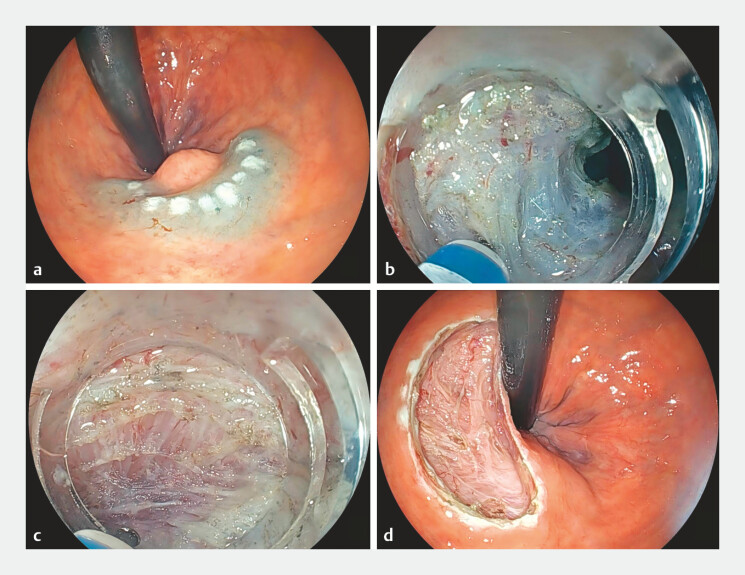
**a** Mucosal marking and submucosal injection. **b** Smooth incision of the circular muscle using the scopeʼs downward-bending capability. **c** The slim profile of the endoscope facilitates smooth dissection within the intermuscular space. **d** The postoperative wound bed.

A 54-year-old man was found to have a 15-mm SEL in the rectum near the anus during a routine physical examination ( Fig. 1 **a** ). Endoscopic ultrasonography confirmed that the lesion originated from the muscularis propria ( Fig. 1 **b** ). Magnetic resonance imaging ruled out distant and lymph node metastasis ( Fig. 1 **c** ). EID was performed with the novel therapeutic ultrathin endoscope ( Fig. 2 and Fig. 3 and Video 1 ). The procedure commenced by utilizing the endoscopeʼs exceptional downward bending angle to smoothly incise the circular muscle with a Dual knife under a forward view. This capability, combined with the endoscopeʼs slim profile, facilitated smoother dissection within the intermuscular space. Furthermore, to overcome the limited field of view, a retroflexed view was employed to thoroughly inspect the anal side of the resection wound. The total procedure time was 50 minutes. Postoperative pathology confirmed a very low-risk gastrointestinal stromal tumor with negative margins.

Endoscopic intermuscular dissection using a novel thin endoscope for gastrointestinal stromal tumors adjacent to the anus.Video 1

The anatomical angle between the anus and the rectum complicates the endoscopic resection of adjacent lesions. EID is challenging for it requires precise dissection within the narrow intermuscular space, unlike the loose submucosal plane 3 4 . The ultrathin endoscope, due to its slim tube and greater bending capability, facilitates access to this plane and maintains excellent visualization and maneuverability within the intermuscular space. This case demonstrates that the therapeutic ultrathin endoscope can serve as an ideal tool for EID, effectively addressing common limitations such as poor visualization and restricted manipulation.

Endoscopy_UCTN_Code_TTT_1AQ_2AC
